# Supplementary material for: Caregiver perceived barriers to the use of micronutrient powder for children aged 6–59 months in Bangladesh
Source: PLoS One. 2021 Dec 2;16(12):e0260773. doi: 10.1371/journal.pone.0260773 (PMC8638897; doi:10.1371/journal.pone.0260773)
Supplement: S1 Table — (PDF) [file pone.0260773.s001.pdf]

S1 Table. Variables and scores used in constructing the infant and child feeding index

**Indicator**	**Age-groups (months)**							
	**6-8**		**9-11**		**12-23**		**24-59**	
	**Value**	**Score**	**Value**	**Score**	**Value**	**Score**	**Value**	**Score**
Currently breastfed	yes	+2	yes	+2	yes	+1	Yes	0
Food groups	1<br>≥2	+1<br>+2	1 or 2<br>≥3	+1<br>+2	2 or 3<br>≥4	+1<br>+2	3 or 4<br>≥ 5	+2<br>+3
Meal frequency	1<br>≥2	+1<br>+2	1 or 2<br>≥3	+1<br>+2	2<br>3<br>≥4	+1<br>+2<br>+3	2<br>3<br>≥4	+1<br>+2<br>+3
