# Supplementary material for: Caregiver perceived barriers to the use of micronutrient powder for children aged 6–59 months in Bangladesh
Source: PLoS One. 2021 Dec 2;16(12):e0260773. doi: 10.1371/journal.pone.0260773 (PMC8638897; doi:10.1371/journal.pone.0260773)
Supplement: Attachment — Submitted filename: Response to Reviewers.doc [file pone.0260773.s002.doc]

Response to reviewers

We appreciate the very thoughtful reviews of the previous version of the manuscript. We have updated the text in response to the reviewers’ queries and feedback. A point-by-point response to each of the reviewers’ comments is included below. We believe these changes have substantially improved the manuscript. We hope you will find this revised manuscript appropriate for publication in PLOS ONE. Many thanks for your consideration.

Journal Requirements:

Please review your reference list to ensure that it is complete and correct. If you have cited papers that have been retracted, please include the rationale for doing so in the manuscript text, or remove these references and replace them with relevant current references. Any changes to the reference list should be mentioned in the rebuttal letter that accompanies your revised manuscript. If you need to cite a retracted article, indicate the article’s retracted status in the References list and also include a citation and full reference for the retraction notice.

**Response:** Thank you for your valuable suggestions. We have checked the reference list and made corrections as per the formatting guidelines. There was an error in referencing ‘ Myatt M. IYCF assessment with small sample surveys: A proposal for a simplified and structured approach’ (#20 in reference list). Details on Infant and Child Feeding Index (ICFI) have been mentioned in ‘Guevarra E, Siling K, Chiwile F, Mutunga M, Senesie J, Beckley W, et al. IYCF assessment with small-sample surveys-A proposal for a simplified and structured approach. Field Exchange 47. 2014 Jul 19:60.’ Therefore, we have replaced the previous reference with this one in the revised manuscript.

When submitting your revision, we need you to address these additional requirements.

1. Please ensure that your manuscript meets PLOS ONE's style requirements, including those for file naming. The PLOS ONE style templates can be found at

[https://journals.plos.org/plosone/s/file?id=wjVg/PLOSOne_formatting_sample_main_body.pdf](https://linkprotect.cudasvc.com/url?a=https%3A%2F%2Fjournals.plos.org%2Fplosone%2Fs%2Ffile%3Fid%3DwjVg%2FPLOSOne_formatting_sample_main_body.pdf&c=E,1,9JiWMs13U6WT-rcHa4bEysfx3yZ4iJLlmZDRoMQHca5udrw18_PEDoLT9SBtnLgNSZfG87is7wQp6l-C1hzFHOV3Q2icTqZRALqqDwI5TAldHVaAqVNZ2mI,&typo=1&ancr_add=1) and

[https://journals.plos.org/plosone/s/file?id=ba62/PLOSOne_formatting_sample_title_authors_affiliations.pdf](about:blank)

**Response:** Thanks. We have checked with the file formatting sample of PLOS ONE and made corrections in the title page. We have also made corrections in file naming as per PLOS ONE style templates.

2. Please ensure you have discussed the limitations of this study within the Discussion section, including any potential bias introduced during sampling and/or data collection.

**Response:** Thank you for your observation. According to your suggestion, we have added a section on ‘Strengths and limitations’ within the Discussion section (Page 17, lines 355-361) in the revised manuscript. Two-stage random sampling technique was applied to select the caregivers of children from households and data collection was done by the trained data collectors, so we assume that no potential bias was introduced during sampling or data collection.

3.  We note that you have indicated that data from this study are available upon request. PLOS only allows data to be available upon request if there are legal or ethical restrictions on sharing data publicly. For information on unacceptable data access restrictions, please see [http://journals.plos.org/plosone/s/data-availability#loc-unacceptable-data-access-restrictions](https://linkprotect.cudasvc.com/url?a=http%3A%2F%2Fjournals.plos.org%2Fplosone%2Fs%2Fdata-availability%23loc-unacceptable-data-access-restrictions.&c=E,1,2s3B3WxaBXzbO5igDJLj77AW-cDs3oaMsEoV0xCy0xoB1fVT1RWnFNg1TiEOFZxCelOt5eyHTHLikBJx49QvRk6EfhaaE2jq5Zl6Ulk1&typo=1&ancr_add=1).

In your revised cover letter, please address the following prompts:

a) If there are ethical or legal restrictions on sharing a de-identified data set, please explain them in detail (e.g., data contain potentially identifying or sensitive patient information) and who has imposed them (e.g., an ethics committee). Please also provide contact information for a data access committee, ethics committee, or other institutional body to which data requests may be sent.

b) If there are no restrictions, please upload the minimal anonymized data set necessary to replicate your study findings as either Supporting Information files or to a stable, public repository and provide us with the relevant URLs, DOIs, or accession numbers. Please see [http://www.bmj.com/content/340/bmj.c181.long](https://linkprotect.cudasvc.com/url?a=http%3A%2F%2Fwww.bmj.com%2Fcontent%2F340%2Fbmj.c181.long&c=E,1,jL7OXy7SM4bxL18XjXnwNJGsQ1qH8J9kgfkOmdod5omk7uHsu0XxxqyY8C6aQTX-hJWHi7YsjIgXP-D3T0pcBO5cFDgcUgjD6sc_O2L8fN2Zlp2COlf4&typo=1&ancr_add=1) for guidelines on how to de-identify and prepare clinical data for publication. For a list of acceptable repositories, please see [http://journals.plos.org/plosone/s/data-availability#loc-recommended-repositories](https://linkprotect.cudasvc.com/url?a=http%3A%2F%2Fjournals.plos.org%2Fplosone%2Fs%2Fdata-availability%23loc-recommended-repositories.&c=E,1,UxFpALQxYDxT1lRFlpWd-ZpCIFb-bLTBSkf3unNBr4v0cYFo2QvAlMuDELsH05NBIH38JZhMkvpYLRVWd0Y_yuI1UeXMYL4s2szm37KVPfBuM9YW-YeLZMV0&typo=1&ancr_add=1).

We will update your Data Availability statement on your behalf to reflect the information you provide.

**Response:** Thanks. We have checked the organization policy and revised the ‘Data Availability statement’ as per your suggestion as follows:

“Data generated from icddr,b’s research can be provided to interested researchers (Recipients) for secondary data analyses upon approval of a Data Licensing Application & Agreement by the icddr,b Data Centre Committee. Interested personnel is recommended to consult this with icddr,b IRB Coordinator Mr. M A Salam Khan (salamk@icddrb.org)”.

4. Please include captions for your Supporting Information files at the end of your manuscript, and update any in-text citations to match accordingly. Please see our Supporting Information guidelines for more information: [http://journals.plos.org/plosone/s/supporting-information](about:blank).

**Response:** Thank you for your observation. We have included caption for our Supporting Information file at the end of the manuscript and update the in-text citation accordingly.

Reviewers' comments:

5. Review Comments to the Author

Reviewer #1: The authors report an ineresting survey of the underlying factors related to the barriers to use of MNP. The survey contains a large sample. I think the results are pretty sond and the data analysis is proper.

**Response:** Thank you very much for your complements.

Reviewer #2: There is no denying that,MNP suppling is the major method for improving nutritional status and reducing anemia. In practice, however, PROTES barriers from the perspective of both supply and demand side.
Based on the demand side, this study analyzed the perception of the caregivers of the childrendemand side and socio-cultural factors.It can provide a basis for improvement in the follow-up health promotion programs.
It is suggested to further summarize and clarify that intervention plans on eliminating the obstacle factors.

**Response:** Thank you for your valuable comments and suggestions. According to your suggestions we have added some suggestive intervention plans based on our findings to eliminate the obstacle factors in discussion section (Pages 17, lines 348-353) of the revised manuscript.
